# Supplementary material for: Plasma γ-Aminobutyric Acid (GABA) Concentrations in Lactating Holstein Cows during Thermoneutral and Heat Stress Conditions and Their Relationships with Circulating Glucose, Insulin and Progesterone Levels
Source: Vet Sci. 2024 Mar 21;11(3):137. doi: 10.3390/vetsci11030137 (PMC10975163; doi:10.3390/vetsci11030137)
Supplement: Supplementary file 1 [file vetsci-11-00137-s001.zip › vetsci-2850392-supplementary.pdf]

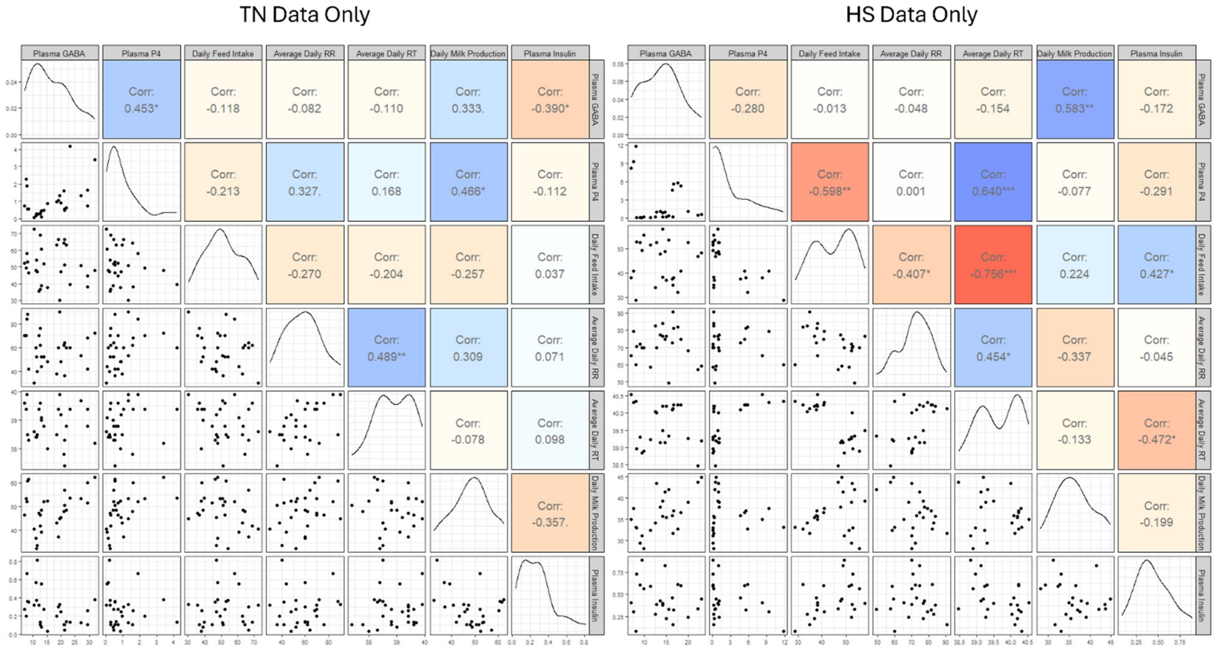

Figure S1. Correlograms showing the correlations between plasma γ-aminobutyric acid (GABA) concentration, plasma progesterone (P4) concentration, daily feed intake, average daily respiration rates (RR), average daily rectal temperatures (RT), daily milk production in kilograms, and plasma insulin concentration for the TN and HS periods separately. The bottom half of the graph shows scatterplots of each variable against the others, and the top half shows the Pearson correlation coefficient for each pairing. A correlation value marked with * shows that the *p*-value is <0.05; *** shows that the *p*-value is <0.01. The diagonal shows a density plot representative of the distribution of each variable.
